# Quality assessment of short videos on health science popularization in China: scale development and validation

**DOI:** 10.3389/fpubh.2025.1640105

**Published:** 2025-09-18

**Authors:** Wenxia Xuan, Kun Tian, Lijie Hao

**Affiliations:** ^1^Shanxi Bethune Hospital, Shanxi Academy of Medical Sciences, Tongji Shanxi Hospital, Third Hospital of Shanxi Medical University, Taiyuan, China; ^2^College of Digital Arts, Communication University of Shanxi, Taiyuan, China; ^3^Faculty of Foreign Languages, Zhejiang Wanli University, Ningbo, China

**Keywords:** health science popularization short videos, short video quality, scale development, scale validation, multimodal theory, exploratory factor analysis, confirmatory factor analysis

## Abstract

**Background:**

Short videos that popularize health science have become essential for disseminating health information and enhancing public health literacy. However, previous research has primarily focused on health information content, with a significant gap in assessing the quality of health science popularization in short videos.

**Methods:**

This study developed a quality assessment scale for the popularization of health science short videos based on multimodal theory, utilizing literature analysis and the creation of custom measurement items. Data were collected from scales completed by 796 residents through online surveys conducted on mobile devices. Both exploratory and confirmatory factor analyses were employed to evaluate the quality of mobile health science popularized short videos.

**Results:**

The results revealed that the quality scale for health science popularization in short videos could be divided into seven dimensions and 22 indicators, each a significant determinant of video quality.

**Conclusion:**

This research provides a more intuitive, reliable, and standardized tool for assessing the quality of health science popularization in short videos. Also, it offers essential guidance for the future design, development, and promotion of short health science popularization videos.

## Introduction

With the increasing prevalence of mobile social media, short mobile videos have become a vital tool for disseminating health-related scientific information around the world (1). According to the “Digital 2023 Global Overview Report,” TikTok, YouTube Shorts, and Instagram Reels have over 2.5 billion monthly active users, and the number of views for health-related content on these platforms alone reaches hundreds of billions (2, 3). People can use these platforms to search for and obtain relevant information and guidance on health, thereby improving their health literacy (4–6). Although short videos have greatly facilitated people’s access to health knowledge, there are still some limitations, such as the authenticity and quality of health information, which influence users’ intentions (7, 8).

In China, short videos have also become an important medium for health education. According to the “2019 Health Science Popularization Videos Insight Report,” among more than 9,000 survey participants, 92.1% indicated that they had watched videos about health science, and 55.3% used these videos to engage with healthy lifestyles (9). This finding highlights the importance of short mobile videos for spreading health knowledge. They change how the public accesses health information, deepen their understanding of health knowledge, and enhance public health literacy (10). Similarly, in the “Guidelines on Establishing a Comprehensive All-Media Health Science Knowledge Publication and Dissemination Mechanism” released in May 2022, the National Health Commission emphasized that various entities, including health departments, internet platforms, and media organizations, should work together to enhance the supply of high-quality health science knowledge to meet the growing health needs of the public (11). These trends demonstrate that the high-quality development and effective dissemination of health science information are highly valued at corporate, societal, and national levels.

Short mobile videos have become a significant platform for disseminating health science information, establishing an interactive bridge between users and this information. However, the quality of short video content for health science popularization varies significantly at this stage because there is a lack of adequate regulatory bodies and scientific evaluation standards. Misleading or false information can even harm users, thus disrupting the usual channels for disseminating health science information and damaging the standard supply and demand relationship between users and health information (12–14). In response to these challenges, some scholars have conducted research from multiple perspectives, including the paths and mechanisms of disseminating health science information (15, 16), users’ needs perception and its impact (17, 18), and the design elements of health science videos (19, 20). Similarly, some scholars have used methods such as DISCERN, JAMA Benchmark, Global Quality Score (GQS), and PEMAT (A/V) to assess the quality of health science popularization short videos (21–23). However, these assessment scales were originally applied to textual materials or long videos, and they lack specificity for 15–60-s multimodal short videos. Some scholars have also used large language models (LLMs) to assess the quality of medical information on short-video platforms, but the technique is still in the experimental stage and has not yet developed into an industry solution that can be directly applied (24). Although these studies have, to some extent, promoted the development of the field, current research lacks dedicated and standardized tools to assess the health science popularization short videos. Furthermore, existing scales lack systematic reliability and validity tests (1, 17). Specifically, the unique ecosystem of short videos presents a theoretically complex definition of quality. This leads to potential discrepancies between evaluation results and users’ actual needs (25), which in turn affects whether the content of health science popularization short videos can meet the core needs of users and limits the potential for high-quality development of health science popularization short videos.

Health science popularization short videos as comprehensive carriers of health information, primarily involving topics, content, presentation forms, and dissemination methods (16). In contrast to legacy media and text-centric formats, health science popularized short videos are marked by fragmented narrative units, high-intensity audiovisual cues, and algorithmically mediated circulation, together producing a complex, reciprocal interactional ecology. While established frameworks such as source credibility, cognitive-load theory, and the Health Belief Model provide a sound conceptual substrate, they are, in isolation, inadequate for apprehending the multidimensionality of user experience in this format. Therefore, this study adopts multimodal theory to investigate how multiple semiotic modes—such as visuals, text, and audio—interact to influence users’ perceptions of video quality (26, 27). In summary, this study aims to systematically develop and validate a multidimensional scale that reflects users’ overall assessment of the quality of short videos focused on health science popularization within this dynamic, multimodal environment. This scale not only provides a theoretical framework grounded in multimodal discourse but also offers a user-centered assessment tool to support the high-quality development of digital health communication and enhance public health literacy in China.

## Literature review

### Evaluation of the quality of short videos on health science

Health science popularization short videos are public health educational videos disseminated through mobile internet platforms to convey scientific health knowledge to the general public. These videos embody authority, rigor, accessibility, and interactivity among users. The quality of the content can significantly impact its dissemination effectiveness (28). Existing research indicates that the quality of health information can affect users’ perceptions and emotional responses to health science videos, influencing their continued usage intentions (17, 20). Thus, the quality of health information plays a crucial role in videos that popularize health science. Evaluation is one of the essential methods for screening health information quality and can play a significant role in the evaluation system (12, 29). Current research on evaluating health information quality primarily focuses on two aspects. On one hand, professional medical personnel discuss the quality of health information and the tools for its evaluation (21). In contrast, the general public assesses the quality of health information (30). Although both approaches evaluate the quality of health information, there is a fundamental difference between them. Medical professionals evaluate and classify health information from a professional medical perspective. This professional assessment and classification can cause cognitive barriers for non-professional, ordinary users, leading to various adverse consequences and impacts (31). Similarly, due to ordinary users’ own circumstances and differences, they may also have different opinions on the quality of health information (25, 30). However, the general public represents the core user group for health science popularization in short videos. Evaluating user experiences with health information directly reflects the quality of videos that popularize health science. Therefore, user evaluations are a critical factor in assessing the quality of health science videos.

Short videos serve as effective carriers for disseminating health information, fulfilling both the application and communicative functions of this information (32). Previous research has explored the impact of health science popularization in short videos in various dimensions, including social behavior (33), security and privacy (34), and artistic and technical aspects (19), uncovering their potential mechanisms of influence. Although these studies have positively contributed to the development of short videos for health science popularization, research on tools for evaluating the quality of these videos has not received sufficient attention. However, the quality of health science popularized in short videos directly affects users’ perception of health information and subsequent usage behaviors (17). Therefore, a practical evaluation of the quality of health science popularization through short videos plays a vital role in developing health education initiatives in our country and enhancing public health literacy.

### Evaluation dimensions of the quality of short videos of health science popularization

Health science popularization short videos are a comprehensive format for presenting health science information involving multiple evaluation dimensions. In past research, scholars have primarily evaluated health information based on three aspects: information characteristics (25), platform design (20, 25), and individual emotions (35). While these dimensions have been effective in assessing health information and have been widely recognized and applied by scholars, there remain issues in evaluating the quality of health information in streaming and short video applications, demonstrating the limitations of a single aspect of the evaluation dimension (12, 36). Given that the quality of health science popularization short videos is an umbrella-like concept involving health information, health services, video design, content format, and user experience, multimodal theory can provide a robust theoretical framework. The rationale for utilizing this theory lies in its ability to elucidate how multiple symbolic resources (such as text, images, sound, and interaction) collaborate to construct a multi-dimensional evaluation system through interaction (26, 27, 37). Compared with prior models that rely on textual criteria or single-mode evaluation tools (such as DISCERN, JAMA Benchmark, and GQS), multimodal theory enables a more nuanced and realistic analysis of how users perceive and evaluate video-based health information (36, 38). Therefore, we utilize a multidimensional perspective to measure and assess quality, taking into account the interdependent coupling effects and configurational relationships among various variables (19, 39). This approach will improve both the accuracy and effectiveness of measuring the quality of short videos that promote health science.

Though existing studies have explored and researched health science popularization information using different theoretical models; however, due to the limitations inherent in each theoretical model, these studies do not comprehensively reflect the quality of health science popularization of short video information (20, 39). To address this issue, this research plans to use multimodal theory as a foundation to delve into different types of literature and explore the dimensions of health science popularization, short video quality from the user perspective, including aspects such as the health blogger’s image, professionalism, video content, interaction, safety, emotion, and practicality.

## Method

### Measure the generation of items

According to the three-step scale development procedure suggested by Churchill and Iacobucci (40), we conducted a systematic review of existing literature covering areas such as health information quality, online community user experience, and short video engagement. Measurement indicators are defined based on the content of the measurement subject and specific domestic conditions. Established scales are used, with appropriate modifications made according to the research context. Drawing from the studies by Ohanian (41), Zhu et al. (42), Bhattacharjee and Sanford (43), Lin and Wang (44), Tang et al. (45), Liu (46), Son and Kim (47), Tian et al. (48), Johnson and Lowe (49), and Cavalo and Brown (50), a scale was developed to measure the quality of mobile health science popularization short videos in China. The scale items cover dimensions related to the health vlogger’s image, professionalism, and information content.

To ensure that the measurement items of the scale adequately cover the measurement factors, this study is based on both theoretical and empirical foundations. A panel of six experts, comprising three professors specializing in digital media within communication studies and three chief physicians experienced in public health education, was convened to assess and enhance the measurement items of the scale. They evaluated each item based on three criteria: relevance (Is the item pertinent to the construct?), clarity (Is the item articulated clearly and without ambiguity?), and comprehensiveness (Does the item pool sufficiently encompass the dimension?). Based on their quantitative ratings and qualitative feedback, some items were either removed due to ambiguity or merged due to conceptual overlap. Guided by the theories of source credibility (41), cognitive load (51), social support (52), and the health belief model (53), six additional items were incorporated: “The professional status of a health blogger makes me feel secure.”; “The clarity and fluency of the health blogger made me feel professional.”; “The health science popularization short videos can clearly explain health science in a short period.”; “The health science popularization short videos have allowed me to meet more like-minded people.”; “The health science popularization short videos are very safe.”; and “The health science popularization short videos play an important and practical role in health.” This represents professionalism, safety, utility, and interactivity, which are essential to addressing the gaps in measurement factors. Finally, the scale items will be reviewed and refined to ensure the correct use of terminology, clarity of language, completeness of content, and the absence of any repetition or omissions. Meanwhile, the bilingual translation-back translation method was performed by two Chinese-English bilingual experts with doctoral degrees to reduce semantic bias, to enhance the consistency of the cross-cultural measurements, and to ensure the applicability of the scales in this study.

Based on the information above, a preliminary scale of 36 items has been developed to assess the quality of mobile health science popularization short videos. A convenience sampling method was used to conduct a pilot survey with 50 residents who regularly watch health science popularization short videos to enhance the scale’s measurement reliability and content validity. The results indicated a Cronbach’s α coefficient of 0.845. Additionally, five experts with backgrounds in communication and medicine were invited to assess the scale, yielding an Inter-Objective Consistency Index (IOC) of approximately 1 (54). These results demonstrate that the scale possesses excellent reliability and validity, thereby warranting the distribution of the formal questionnaire (Table 1).

**Table 1:** Indicators for measuring the quality of short health science videos and literature sources.

Dimension	Measurement indicators	Sources
Health blogger image	Health bloggers who are well-groomed and fit make me feel approachable.	Ohanian (41); Self-paced questionnaire
	The excellent image qualities of health bloggers appeal to me.	
	Health bloggers’ formal attire makes me feel authoritative.	
	The professional status of a health blogger makes me feel secure.	
Health blogger professionalism	Health bloggers’ descriptions of expertise can reflect a vast knowledge base.	Zhu et al. (42); Bhatacherjee and Sanford (43)
	Health bloggers can be proactive and effective in coming up with solutions to health problems.	
	Health bloggers articulate reliable health science knowledge.	
	Health bloggers can objectively spread health science knowledge without distorting and exaggerating facts.	
	The clarity and fluency of the health blogger make me feel professional.	
	The health blogger can get back to me promptly about my health issues.	
Health science short video content	The health science popularization short video presentation is new and exciting enough to get my attention.	Lin and Wang (44); Tang et al. (45); Self-paced questionnaire
	The health science popularization short videos are informative and meet my needs.	
	The content of the health science popularization short videos is authentic and reliable, which gives me peace of mind.	
	The content of the short videos on health science popularization is straightforward, providing me with a comprehensive understanding of health.	
	The popularization of health science short videos provides practical health knowledge and skills.	
	The health science popularization short videos can clearly explain health science in a short period.	
Health science short video interaction	I can ask my health questions anytime through the health science popularization short videos.	Liu (46); Self-paced questionnaire
	The popularization of health science short videos provides me with more avenues to learn about and understand health.	
	I can use health science popularization short videos to share with my friends anytime.	
	The health science popularization short videos have allowed me to meet more like-minded people.	
	The health science popularization short videos have helped me set health goals.	
Health science short video safety	The health science popularization short videos do not provide personal information for use by others.	Son and Kim (47); Tian et al. (48)
	The popularization of short health science videos will prevent others from accessing my private information.	
	The health science popularization short videos will protect my personal information.	
	The health science popularization short videos have perfect self-privacy permission protection.	
	The health science popularization short videos are very safe.	
Health science short video emotion	The health science popularization short videos can give me peace of mind.	Johnson and Lowe (49); Tang et al. (45)
	The health science popularization short videos can help me beat the odds.	
	The health science popularization short videos can cheer me up.	
	The health science popularization short videos can evoke an emotional connection with others.	
	The health science popularization short videos can ease my anxiety.	
Health science short video use	The health science popularization short videos have provided me with a positive and practical health program.	Cavalo and Brown (50); Self-paced questionnaire
	The popularization of health science short videos explains the importance of health science knowledge in health issues.	
	The health science popularization short videos detail how to reach health goals through health literacy.	
	The popularization of health science short videos highlights the need for health science knowledge to improve health problems.	
	The health science popularization short videos play an important and practical role in health.	

The formal questionnaire is divided into two parts. The first part collects sociodemographic characteristics, including gender, age, occupation, and income. The second part consists of the measurement items rated on a 5-point Likert scale, where 1 represents “strongly disagree,” 2 represents “disagree,” 3 represents “neutral,” 4 represents “agree,” and 5 represents “strongly agree.”

### Data collection and sample characteristics

This study’s survey was created using the Questionnaire Star system and distributed via mobile phones. Online distribution offers several advantages, such as not being limited to a single geographical location, low costs, and rapid data collection (55). To increase participants’ willingness to engage, those who completed the survey were randomly rewarded with red envelopes containing monetary rewards ranging from 2 to 5 yuan. Currently, second- and third-tier cities in China are the centers of city development and population flow (56). To consider the universality of the sample and the issues arising from regional differences (such as information literacy and health literacy), all of our respondents are from second- and third-tier cities. The survey collected 870 responses, of which 74 were excluded due to respondents not watching health science popularization short videos or completing the survey in less than 3 min, leaving 796 valid responses, yielding a validity rate of 91.4%. The sample size has fully reached the standard of 10 times the number of observational indicators ( $N_{\min}=10*k$ ) and root mean square error of approximation (RMSEA)-based power analysis for exploratory and confirmatory analyses $(N=\frac{X_{1}^{2}-\alpha ,\mathrm{df}-X_{1}^{2}-\beta ,\mathrm{df}}{\mathrm{df}\cdot (\in_{A}^{2}-\in_{0}^{2})}),$ allowing for statistical analysis to be conducted (57, 58). The total sample (S = 796) was randomly divided into two parts: Sample 1 (S1 = 398) was used for exploratory factor analysis, and Sample 2 (S2 = 398) for confirmatory factor analysis.

In terms of demographic characteristics of the sample, males accounted for 48.2%, and females 51.8%. The age distribution was as follows: 18–35 years old represented 23.5%, 36–50 years old represented 40.3%, 50–65 years old represented 27.4%, and over 65 years old represented 8.8%. Professionally, the sample was predominantly composed of students, private sector employees, self-employed individuals, civil servants, and public sector employees, accounting for 66.4%. A high school diploma or college degree was the most common regarding educational attainment, representing 69.7% of the sample. As for income, most respondents earned between 3,001 and 7,000 renminbi (RMB), accounting for 52.3% of the analysis.

## Results

### Exploratory factor analysis and results

An exploratory factor analysis was conducted on the initial scale items to explore the structural dimensions of health science popularization of short video quality. Using Sample 1 (S1 = 398), the 36 scale items were subjected to the Kaiser–Meyer–Olkin (KMO) test and Bartlett’s test of sphericity. The results indicated a KMO value of 0.818, more than the acceptable threshold of 0.70, and Bartlett’s test of sphericity resulted in *p* < 0.001, which is less than 0.05, suggesting that the items are suitable for factor analysis. Principal component analysis and varimax rotation were used to obtain factor loadings, with eigenvalues greater than 1 as the criterion for factor extraction. Items with commonalities less than 0.5 (40), factor loadings below 0.5 (57), cross-loadings greater than 0.4 (59), and inconsistent with the meaning of the associated factor were excluded. Additionally, a Corrected Item-Total Correlation (CITC) was conducted to screen the items, and items with a CITC coefficient less than 0.4 were removed (40). Using the item-by-item deletion method, first delete items with low factor loadings, high cross-factor loadings, or low commonality, and then gradually optimize the model structure (60). Through six rounds of factor analysis, 14 items that did not meet the requirements were eliminated (Table 2), leaving 22 items. These extracted seven factors with eigenvalues greater than 1, explaining 79.08% of the total variance, which exceeds the 60% standard (61), indicating that these factors adequately represent most of the information of the items. The reliability test results indicated that Cronbach’s α coefficients for the seven factors were above 0.70 (62), which is considered acceptable. Overall, the scale’s reliability is reliable, and its dimensional structure is relatively stable (Table 3).

**Table 2:** Eliminate items in each round.

Round	Item	Factor loading/cross-factor loadings/commonality
1	Health bloggers who are well-groomed and fit make me feel approachable.	0.267; 0.238; 0.201
	Health bloggers can be proactive and effective in coming up with solutions to health problems	0.430; 0.240; 0.317
	Health bloggers articulate reliable health science knowledge.	0.482; 0.184; 0.318
2	The content of the health science popularization short videos is authentic and reliable, which gives me peace of mind.	0.436; 0.134; 0.256
	The health science popularization short videos can cheer me up.	0.408; 0.353; 0.396
	The popularization of short health science videos will prevent others from accessing my private information	0.480; 0.261; 0.366
3	The content of the short videos on health science popularization is straightforward, providing me with a comprehensive understanding of health.	0.560; 0.187; 0.378
	The health blogger can get back to me promptly about my health issues.	0.454; 0.378; 0.402
4	The health science popularization short videos can evoke an emotional connection with others.	0.468; 0.227; 0.357
	The health science popularization short videos have allowed me to meet more like-minded people.	0.573; 0.117; 0.374
5	The popularization of health science short videos provides practical health knowledge and skills.	0.494; 0.338; 0.397
	The health science popularization short videos have helped me set health goals.	0.499; 0.192; 0.428
6	The health science popularization short videos detail how to reach health goals through health literacy.	0.456; 0.398; 0.458
	The popularization of health science short videos highlights the need for health science knowledge in addressing health problems.	0.684; 0.424; 0.625

**Table 3:** Results of exploratory factor analysis.

Factor	Items	Communality	Factor loading	Eigenvalue	Cumulative explained variance/%	Cronbach’s α
F_1_ Safety	The health science popularization short videos will protect my personal information.	0.827	0.908	3.814	17.335	0.909
	The health science popularization short videos do not provide personal information for use by others.	0.780	0.869			
	The health science popularization short videos have perfect self-privacy permission protection.	0.783	0.880			
	The health science popularization short videos are very safe.	0.805	0.878			
F_2_ Professionalism	Health bloggers’ descriptions of expertise can reflect a vast knowledge base.	0.743	0.825	3.123	31.531	0.811
	Health bloggers can objectively spread health science knowledge without distorting and exaggerating facts.	0.715	0.814			
	The clarity and fluency of the health blogger make me feel professional.	0.791	0.877			
F_3_ Utility	The health science popularization short videos have provided me with a positive and practical health program.	0.800	0.874	2.298	43.097	0.875
	The popularization of health science short videos explains the importance of health science knowledge in health issues.	0.821	0.900			
	The health science popularization short videos plays an important and practical role in health.	0.808	0.888			
F_4_ Emotionality	The health science popularization short videos can give me peace of mind.	0.799	0.884	2.545	53.542	0.845
	The health science popularization short videos can help me beat the odds.	0.755	0.852			
	The health science popularization short videos can ease my anxiety.	0.762	0.869			
F_5_ Quality	The health science popularization short video presentation is new and exciting enough to get my attention.	0.799	0.870	2.009	63.084	0.878
	The health science popularization short videos are informative and meet my needs.	0.810	0.874			
	The health science popularization short videos can clearly explain health science in a short period.	0.831	0.902			
F_6_ Image	The excellent image qualities of health bloggers appeal to me.	0.788	0.881	1.993	72.143	0.866
	Health bloggers’ formal attire makes me feel authoritative.	0.811	0.896			
	The professional status of a health blogger makes me feel secure.	0.790	0.875			
F_7_ Interactivity	I can ask my health questions anytime through the health science popularization short videos.	0.810	0.890	1.526	79.081	0.867
	The popularization of health science short videos provides me with more avenues to learn about and understand health.	0.802	0.888			
	I can use health science popularization short videos to share with my friends anytime.	0.771	0.858			

Based on the content and characteristics of the items encompassed by the seven aggregated factors, the seven aggregated factors are named as follows: F1 Safety, F2 Professionalism, F3 Utility, F4 Emotionality, F5 Quality, F6 Image, and F7 Interactivity. Specifically, F1 Safety includes four items that reflect the ability of health science popularization short videos to protect users’ personal privacy and safety. F2 Professionalism comprises three items that capture the professional attributes of health vloggers within health science popularization short videos. F3 Utility contains three items designed to measure the practicality and importance of popularizing short videos among users of health science. F4 Emotionality includes three items that demonstrate how health science popularization short videos meet users’ emotional needs. F5 Quality consists of three items that assess the impact of the content quality of health science popularization short videos on users. F6 Image encompasses three items that reflect the influence of the image and demeanor of health vloggers on users of health science popularization short videos. F7 Interactivity includes three items that illustrate the effect of health science popularization and short videos on user information and social interaction.

### Confirmatory factor analysis and results

The Amos 23.0 software was utilized to conduct confirmatory factor analysis on the data from Sample 2 (S2 = 398), primarily because this software is known for reducing bias in estimations and providing more accurate computational results within structural equation modeling. The model’s fit was assessed using various indices to determine how well the model developed through exploratory factor analysis matches the observed data. Following the recommendations of Hair et al. (62), this study selected the fit indices and criteria shown in Table 4 for evaluation. Global fit was satisfactory (Comparative Fit Index (CFI) = 0.957; RMSEA = 0.048). Consistent with parsimony guidelines, we inspected modification indices (MI) and found no values exceeding 15 (maximum = 12.75). This indicates negligible local misfit; therefore, the theorized factor structure remained unchanged in accordance with parsimony recommendations (63). The results indicate that the model meets the established criteria, demonstrating satisfactory model fit.

**Table 4:** Overall model fit results.

Criterion	Fitness threshold	Value of the criterion	Result
Absolute Fit Indices			
CMIN/DF	<3 (Good), < 2(Excellent)	1.910	Excellent
RMR	<0.05	0.049	Good
GFI	>0.90	0.926	Good
AGFI	>0.90	0.901	Good
RMSEA	<0.08 (Good), <0.05 (Excellent)	0.048	Excellent
Incremental Fit Indices			
NFI	>0.9 0(Good), >0.9 5(Excellent)	0.914	Good
IFI	>0.90 (Good), >0.95 (Excellent)	0.957	Excellent
TLI	>0.90 (Good), >0.95 (Excellent)	0.947	Good
CFI	>0.90 (Good), >0.95 (Excellent)	0.957	Excellent
Parsimony Normed Fit Index			
PNFI	>0.50	0.744	Good
PCFI	>0.50	0.779	Good

CMIN/DF, Chi-Square Minimum Discrepancy/Degrees of Freedom; RMR, Root Mean Square Residual; GFI, Goodness-of-Fit Index: AGFI, Adjusted Goodness-of-Fit Index; RMSEA, Root Mean Square Error of Approximation; NFI, Normed Fit Index; IFI, Incremental Fit Index; TLI, Tucker-Lewis Index; CFI, Comparative Fit Index; PNFI, Parsimony Normed Fit Index; PCFI, Parsimony Comparative Fit Index.

This study conducted a reliability and validity test of the scale, including composite reliability, convergent validity, discriminant validity, and criterion validity. In this study, the composite reliability for the seven factors of the scale ranged from 0.766 to 0.874, all above the 0.70 threshold, indicating strong reliability of the scale (64). The evaluation of convergent validity primarily considered the following criteria: standardized factor loadings greater than 0.50 (65); composite reliability (CR) greater than 0.70 (64); and average variance extracted (AVE) greater than 0.50 (66). Meeting these conditions suggests good convergent validity for the scale. The results from the confirmatory factor analysis (Table 5) show that the standardized factor loadings for the seven factors in this study’s scale are all above 0.70, with their composite reliability values also exceeding 0.70, and the AVE values are all above 0.50, indicating excellent convergent validity.

**Table 5:** Validation reliability and convergent validity results.

Factor	Items	Factor loading	CR	AVE
Safety	The health science popularization short videos will protect my personal information.	0.768	0.862	0.610
	The health science popularization short videos do not provide personal information for use by others.	0.812		
	The health science popularization short videos have perfect self-privacy permission protection.	0.700		
	The health science popularization short videos are very safe.	0.837		
Professionalism	Health bloggers’ descriptions of expertise can reflect a vast knowledge base.	0.828	0.811	0.590
	Health bloggers can objectively spread health science knowledge without distorting and exaggerating facts.	0.758		
	The clarity and fluency of the health blogger made me feel professional.	0.714		
Utility	The health science popularization short videos have provided me with a positive and practical health program.	0.750	0.781	0.543
	The popularization of health science short videos explains the importance of health science knowledge in health issues.	0.730		
	The health science popularization short videos plays an important and practical role in health.	0.731		
Emotionality	The health science popularization short videos can give me peace of mind.	0.750	0.794	0.563
	The health science popularization short videos can help me beat the odds.	0.730		
	The health science popularization short videos can ease my anxiety.	0.731		
Quality	The health science popularization short video presentation is new and exciting enough to get my attention.	0.775	0.874	0.699
	The health science popularization short videos are informative and meet my needs.	0.887		
	The health science popularization short videos can clearly explain health science in a short period.	0.749		
Image	The excellent image qualities of health bloggers appeal to me.	0.738	0.766	0.521
	Health bloggers’ formal attire makes me feel authoritative.	0.709		
	The professional status of a health blogger makes me feel secure.	0.719		
Interactivity	I can ask my health questions anytime through the health science popularization short videos.	0.719	0.767	0.524
	The popularization of health science short videos provides me with more avenues to learn about and understand health.	0.727		
	I can use health science popularization short videos to share with my friends anytime.	0.725		

If the correlation coefficient between a factor and all other factors is less than the square root of its AVE, then the discriminant validity is considered satisfactory (66). As shown in Table 6, the square root of the AVE values for the seven factors in this scale is higher than the correlation coefficients between each factor and all other factors. Therefore, the scale exhibits excellent discriminant validity.

**Table 6:** Results of divergent validity analysis.

Dimension	Safety	Professionalism	Utility	Emotionality	Quality	Image	Interactivity
Safety	**0.781**						
Professionalism	0.106	**0.768**					
Utility	0.131	0.229	**0.763**				
Emotionality	0.479	0.269	0.222	**0.750**			
Quality	0.458	0.432	0.323	0.532	**0.836**		
Image	0.406	0.334	0.187	0.425	0.478	**0.721**	
Interactivity	0.341	0.460	0.288	0.468	0.585	0.536	**0.723**

Values on the diagonal are the square root of the mean extracted variance, and values below the diagonal are the correlation coefficients.

### Predictive validity analysis and results

Predictive validity is used to assess the extent to which a scale can predict outcomes related or unrelated to the measured construct (67). In the study of short videos, empirical evidence demonstrates that users’ evaluations of content, interaction, entertainment, and privacy security can influence their satisfaction and willingness to continue using the platform (68–70). Similarly, health science popularization short videos, as a type of short video, encompass inherent characteristics of short videos (71). Therefore, it is reasonable to hypothesize that users’ perceptions of various factors, such as content and format, in health science popularization short videos will also affect their satisfaction and willingness to continue using them. Additionally, health science information is a primary method and crucial pathway for enhancing public health literacy (72). Xuan et al. (73) argue that the content of health science information influences people’s perceptions of health knowledge, trust, and willingness to continue using it. Similarly, the quality of health science information directly impacts people’s satisfaction, thereby affecting their usage intentions (33, 74). It is evident that users’ perceptions of health science popularization short videos are fundamentally perceptions of health information content and quality, which influence users’ emotional responses and usage intentions.

In summary, this study selects user satisfaction and continued usage intention as criterion variables for correlational validity (75). The aforementioned correlation criteria were measured using a 5-point Likert scale, where 1 represents “strongly disagree,” 2 represents “disagree,” 3 represents “neutral,” 4 represents “agree,” and 5 represents “strongly agree.” The questionnaire content was adapted to align with Chinese linguistic expressions and the specific context of this study. A total of 302 questionnaires were distributed both online and offline, with 54 incomplete or invalid responses excluded. The final sample consisted of 248 valid questionnaires used for criterion validity analysis. All items in the questionnaire had Cronbach’s *α* coefficients greater than 0.70.

This study employed correlation and regression analyses to further assess the criterion-related validity of the health science popularization short video quality scale. The correlation analysis results indicated that the seven dimensions of the health science popularization short video quality scale positively correlate with two criterion constructs: user satisfaction and continued usage intention (*p* < 0.05). All pairwise correlation coefficients between variables were greater than 0. (Table 7). The regression analysis used factors affecting the quality of health science popularized short videos as independent variables, while the two criterion constructs served as dependent variables. The results of Table 8 showed that the regression models for the impact factors on user satisfaction [R^2^ = 0.540, *F*(7, 390) = 40.283, *p* < 0.001] and continued usage intention [R^2^ = 0.625, *F*(7, 390) = 57.243, *p* < 0.001] were statistically significant, both indicating adequate explanatory power with R^2^ values exceeding 50%. Notably, the scale measuring the quality of health science popularization in short videos demonstrates stronger explanatory power for users’ continued usage intentions. Additionally, the Durbin–Watson values for satisfaction (1.178) and continued usage intention (1.053) suggest positive autocorrelation in the residuals, which clears the potential issue with the model. In all, based on the above indices, these two regression models demonstrate acceptable model fit. Besides, all the VIF values of the seven predictors were less than 5, indicating no multicollinearity issues among the independent variables (76).

**Table 7:** Correlation analysis results.

Factor	S	P	U	E	Q	I	Inter	Sat	CI
S	1								
P	0.190^**^	1							
U	0.196^**^	0.325^**^	1						
E	0.286^**^	0.179^**^	0.192^**^	1					
Q	0.366^**^	0.360^**^	0.354^**^	0.356^**^	1				
I	0.294^**^	0.270^**^	0.274^**^	0.173^**^	0.330^**^	1			
Inter	0.145^*^	0.367^**^	0.355^**^	0.170^**^	0.408^**^	0.378^**^	1		
Sat	0.380^**^	0.424^**^	0.459^**^	0.407^**^	0.528^**^	0.445^**^	0.528^**^	1	
CI	0.442^**^	0.446^**^	0.493^**^	0.366^**^	0.609^**^	0.498^**^	0.548^**^	0.809^**^	1

S, Safety; P, Professionalism; U, Utility; E, Emotionality; Q, Quality; I, Image; Inter, Interactivity; Sat, Satisfaction; CI, Continued usage intention. *p < 0.05; **p < 0.01.

**Table 8:** The results of regression model statistics for user satisfaction and continued usage intention.

Model	R	R^2^	Adjusted R^2^	Standard error	Durbin–Watson	F	Sig
Sat	0.735^a^	0.540	0.527	1.119	1.178	40.283	0.000
CI	0.791^a^	0.625	0.614	1.011	1.053	57.243	0.000

Sat, Satisfaction; CI, Continued usage intention.

**Figure 1:**
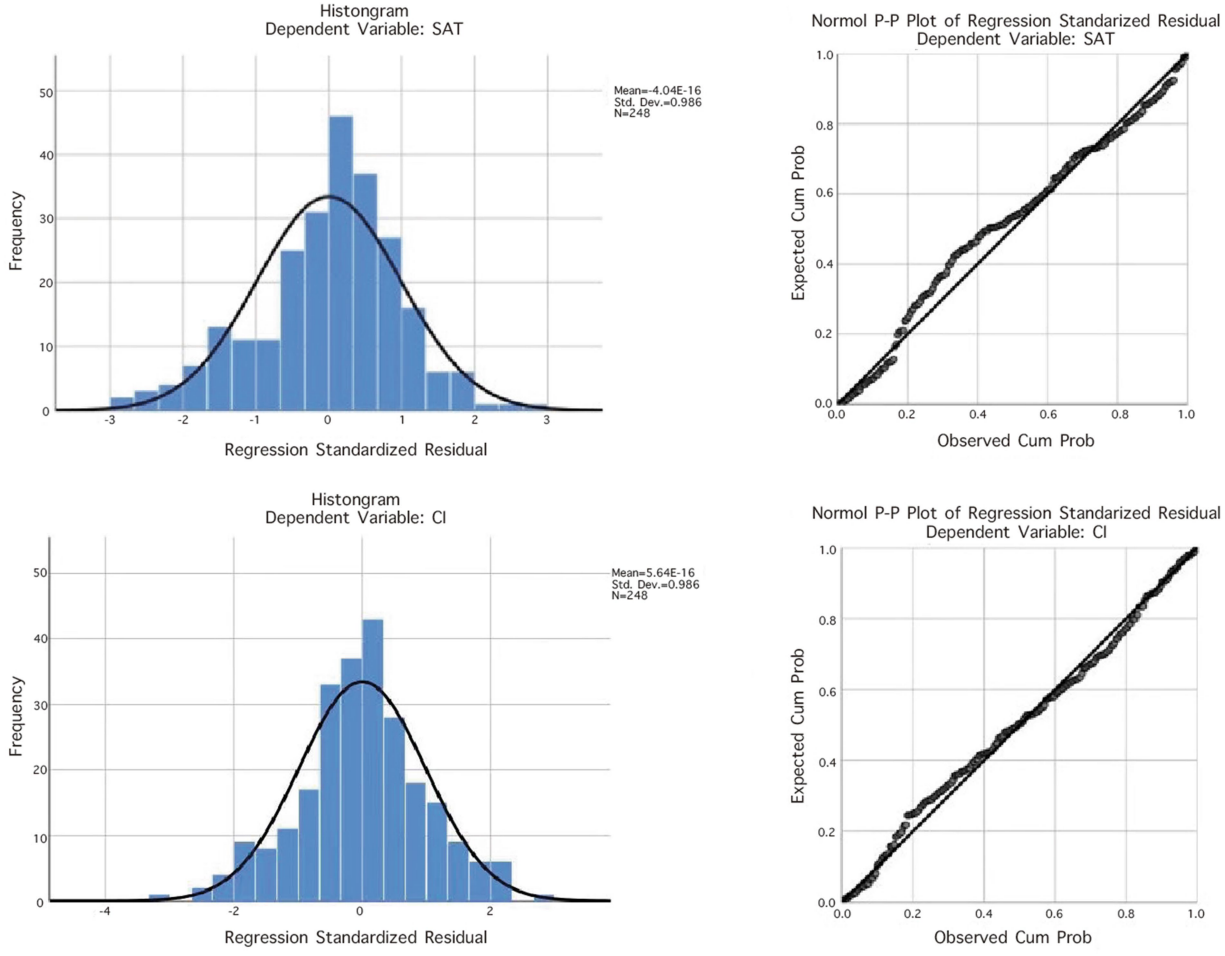
Residual histogram and residual plot.

The regression models for each dimension of the quality of health science popularization short videos on user satisfaction and continued usage intention are all significant and positively influence these variables (Table 9). Analysis of the residual histograms and residual plots for the models of user satisfaction and continued usage intention shows that the mean residuals are close to zero, with standard deviations close to one. The standardized residuals are distributed around the zero value, exhibiting a roughly symmetrical pattern above and below the zero line, indicating that the linear regression meets the normality condition, and the homoscedasticity and independence conditions of the data are met (Figure 1). To further validate the robustness of the models, the interaction effects of the seven factors of the quality of health science popularization short videos on user satisfaction and continued usage intention were examined. The results indicate that the interaction terms of the seven dimensions significantly influence user satisfaction (β = 0.615, t = 12.218, *p* < 0.01) and continued usage intention (β = 0.670, t = 14.161, p < 0.01). Therefore, the health science short popularization video quality scale demonstrates good predictive validity.

**Table 9:** Regression analysis results.

Factor	Satisfaction	Continued usage intention
	β (Standardized coefficient)	β (Standardized coefficient)
Safety	0.136^**^	0.187^***^
Professionalism	0.119^*^	0.111^*^
Utility	0.171^**^	0.182^***^
Emotionality	0.188^***^	0.097^*^
Quality	0.162^**^	0.256^***^
Image	0.146^**^	0.175^***^
Interactivity	0.251^***^	0.229^***^

***p < 0.001; **p < 0.01; *p < 0.05.

In summary, after rigorous steps in scale development and validation, a scale for assessing the quality of health science popularization short videos has been established. Additionally, through a series of reliability and validity tests, the reliability, stability, and scientific validity of this scale have been ensured.

## Discussion

Based on a literature review and grounded in multimodal theory, this study developed a scale to assess the quality of health science popularization short videos in China. This paper provides significant theoretical insights into the study of health science popularization short video quality and offers practical guidance for the design, innovation, and development of health science popularization short videos.

### Theoretical implications

First, this study operationalizes the abstract concept of health science by popularizing short videos for quantitative measurement. The project has expanded the scope of related research in this field with new research scenarios. Specifically, the research developed and validated a scale for assessing the quality of health science popularization short videos, categorizing and summarizing the various factors influencing their quality. It more accurately delineates the dimensions of health science popularization, short video quality, and their interrelations, effectively supporting and supplementing existing research on it. For instance, in assessing the quality of health science popularization short videos, it is necessary not only to evaluate aspects such as the content, quality, and interaction (16, 17, 19), but also to assess the portrayal of characters, their traits, and their professional performance within the videos. In particular, integrating seven dimensions to assess the relationship between user behavior and the quality of health education videos extends existing research frameworks and integrates theory. This approach allows for a more comprehensive interpretation of video quality. Consequently, the scale constructed can measure and reflect the quality of health science popularization short videos more intuitively and reliably, providing a theoretical framework and measurement tools for subsequent related research.

Second, based on multimodal theory, this study precisely categorizes the content of health science popularization short videos, expanding into various modalities such as characters, emotions, safety, and interaction. This approach enhances the application of multimodal theory in health science popularization short videos, strengthens the structural relationship between theory and practice, and provides scientific theoretical guidance for practical applications. Additionally, the research confirms the importance of different independent informational modalities in the construction and transmission process of health science popularization short videos. It also highlights the significance of interaction and integration between different modalities in enhancing the quality of information. Specifically, the factors that affect the quality of health science popularization include short video information, the positive image of health vloggers, and their professionalism, all of which can effectively attract audiences. Safe and practical health information provides users with a positive emotional experience, security, and practicality. Effective interaction and sharing can enhance communication among users and between users and health vloggers, not only fulfilling the social needs of the user community but also efficiently spreading health information and promoting the improvement of public health literacy (33, 74). It is evident that relatively independent factors in health science popularization short videos can significantly influence the quality of the videos and user engagement. The interplay and superposition of these factors not only play a crucial role in determining the quality of health science popularization short videos but also have a lasting impact on users’ emotions and willingness to use such content. Therefore, research has confirmed that in the new media environment, information is inextricably linked to its presentation and the medium itself, further confirming the importance of multimodality. Meanwhile, the research results emphasize the necessity of transcending text-centric assessment models and highlight the importance of interactions between different symbolic modes in shaping user perceptions and subsequent intentions. The interactive relationships among these factors can provide a more objective and accurate reflection of the quality of short videos focused on health science popularization. In summary, these findings further enrich the implications of multimodal theory in assessing the popularity of short video quality in health science.

### Practical implications

The instrument validated here operationalizes the elusive construct of quality, converting it into implementable tactics for stakeholders across the health science popularization short video ecosystem. For digital health content creators, the seven dimensions schema functions as an empirically anchored resource for diagnostic self-assessment and for tuning content-optimization workflows. By attending—systematically—to facets spanning professional behavior and utility through to user emotional resonance and interactive affordances, creators can more credibly create video information and magnify the translational reach of their messages. For platform operators and policymakers, the scale furnishes a shared standard for quality assurance and for iteratively refining content and promotion algorithms. It specifies empirical criteria for elevating content that scores highly on professionalism and safety, thereby stabilizing the information environment and underpinning sectoral guidelines aimed at curbing misinformation. Additionally, for public-health educators and researchers, the scale operates as a standardized instrument for evidence-based curation and for executing large-scale empirical design of the digital-health media landscape. Applied in practice, the instrument can steer the design of targeted public-health interventions and dissemination strategies. Taken together, these uses advance a multi-stakeholder, system-level approach to improving systematically the quality and effectiveness of digital health communication within today’s media ecology.

### Research limitations and perspectives

The chief limitation of this study concerns the representativeness of its sampling frame. Empirical evidence was drawn mainly from residents of China’s second- and third-tier municipalities. As a result, perspectives from first-tier megacities and extensive rural constituencies are underrepresented—contexts in which media-use ecologies, health-information access, and health-literacy profiles plausibly differ substantially. Future studies should undertake multi-site, demographically diverse sampling to re-examine the scale’s properties and strengthen external validity and generalizability. Second, the research design was exclusively quantitative. While appropriate for building a psychometrically reliable instrument, this strategy obscures the subtleties of user experience in specific scenarios. Accordingly, subsequent inquiries would benefit from a mixed-methods design that integrates the current scale with targeted qualitative exploration. Finally, instrument development and validation were conducted within a specifically Chinese cultural milieu. The instrument’s cross-cultural applicability remains empirically unresolved. Future research should adapt and revalidate the instrument across varied cultural contexts to assess its global utility.

## Conclusion

This study targets a long-standing lacuna in health communication by constructing and validating a purpose-built, comprehensive scale for evaluating health science popularity in short videos. Research indicates that the quality of health science popularization short videos is structured along 7 principal dimensions and 22 indicators—spanning safety, professionalism, practicality, emotional appeal, content integrity, visual presentation, and interactivity. Taken in the round, the research result yields a more rigorous, standardized evaluative rubric for effectiveness—and, no less importantly, delineates implementable levers through which creators, platform operators, and regulators can lift video quality and deepen user engagement. Accordingly, the validated instrument functions as a cornerstone for systematic quality assurance, a spur to content innovation, and an anchor for digital health-education benchmarks—thereby advancing public-health literacy and amplifying the beneficial reach of online health information in today’s digital environment.

## Data Availability

Data will be made available upon reasonable request to the corresponding author KT, 170222663@qq.com.
